# Genetic Silencing of Fatty Acid Desaturases Modulates α-Synuclein Toxicity and Neuronal Loss in Parkinson-Like Models of *C. elegans*

**DOI:** 10.3389/fnagi.2019.00207

**Published:** 2019-08-06

**Authors:** Malabika Maulik, Swarup Mitra, Ajiel Mae Basmayor, Brianna Lu, Barbara E. Taylor, Abel Bult-Ito

**Affiliations:** ^1^Department of Chemistry and Biochemistry, University of Alaska Fairbanks, Fairbanks, AK, United States; ^2^Biomedical Learning and Student Training (BLaST) Program, University of Alaska Fairbanks, Fairbanks, AK, United States; ^3^Department of Oral Biology, University at Buffalo, The State University of New York at Buffalo, Buffalo, NY, United States; ^4^Program in Neuroscience, Department of Pharmacology and Toxicology, The Research Institution on Addiction, The State University of New York at Buffalo, Buffalo, NY, United States; ^5^Department of Biology and Wildlife, University of Alaska Fairbanks, Fairbanks, AK, United States; ^6^Department of Biological Sciences and College of Natural Sciences and Mathematics, California State University at Long Beach, Long Beach, CA, United States

**Keywords:** *C. elegans*, fat, Parkinson’s disease, Δ9 desaturase, α-synuclein, dopamine

## Abstract

The molecular basis of Parkinson’s disease (PD) is currently unknown. There is increasing evidence that fat metabolism is at the crossroad of key molecular pathways associated with the pathophysiology of PD. Fatty acid desaturases catalyze synthesis of saturated fatty acids from monounsaturated fatty acids thereby mediating several cellular mechanisms that are associated with diseases including cancer and metabolic disorders. The role of desaturases in modulating age-related neurodegenerative manifestations such as PD is poorly understood. Here, we investigated the effect of silencing Δ9 desaturase enzyme encoding *fat-5* and *fat-7* genes which are known to reduce fat content, on α-synuclein expression, neuronal morphology and dopamine-related behaviors in transgenic PD-like models of *Caenorhabditis elegans* *(C. elegans)*. The silencing of the *fat-5* and *fat-7* genes rescued both degeneration of dopamine neurons and deficits in dopamine-dependent behaviors, including basal slowing and ethanol avoidance in worm models of PD. Similarly, silencing of these genes also decreased the formation of protein aggregates in a nematode model of PD expressing α-synuclein in the body wall muscles and rescued deficits in resistance to heat and osmotic stress. On the contrary, silencing of *nhr-49* and *tub-1* genes that are known to increase total fat content did not alter behavioral and pathological endpoints in the PD worm strains. Interestingly, the genetic manipulation of all four selected genes resulted in differential fat levels in the PD models without having significant effect on the lifespan, further indicating a complex fat homeostasis unique to neurodegenerative pathophysiology. Overall, we provide a comprehensive understanding of how Δ9 desaturase can alter PD-like pathology due to environmental exposures and proteotoxic stress, suggesting new avenues in deciphering the disease etiology and possible therapeutic targets.

## Introduction

Parkinson’s disease (PD) is one of the most prevalent age-related neurodegenerative disorders causing motor impairments and cognitive dysfunction (Beitz, 2014). PD usually results in degeneration of dopaminergic neurons within the substantia nigra (SN) region of the brain. Such degeneration is often correlated with the formation of protein aggregates called Lewy bodies (Michel et al., 2013). Although treatments are available to reduce individual symptoms such as motor deficits, there are currently no preventive therapies that can target and lessen PD progression.

Both genetic and environmental factors have been implicated in the pathogenesis of PD (Elbaz and Tranchant, 2007; Shulman et al., 2011). The α-synuclein (α-syn) gene SNCA was the first to be associated with PD (Polymeropoulos et al., 1997). Both mutations in the SNCA gene and increased copy number results in PD pathology, which is typically characterized by the formation of α-synuclein protein aggregates (Spillantini, 1999). Further, the A53T mutation, which is a substitution of threonine for alanine at position 53 of SNCA gene, is linked with the autosomal dominant form of PD (Polymeropoulos et al., 1997). Exposure to environmental toxins such as rotenone, a broad-spectrum insecticide may lead to mitochondrial dysfunction and oxidative stress causing dopaminergic cell loss in the SN, a key PD-like pathophysiology (Goldman et al., 2012). Aging, which is often accompanied by alterations in metabolic and cellular profiles is known to precipitate the effects of these genetic and environmental factors leading to progression of the disorder (Hindle, 2010; Cooper et al., 2015).

Lipids found abundantly in brain are crucial for development and neural communications (Barber and Raben, 2019). Disruption of lipid metabolism is often linked with neurodegenerative disorders, including Parkinson’s (Ashrafi, 2007; Maulik et al., 2019). Proteins associated with lipid metabolism such as α-synuclein have been identified as risk factors for PD (Fanning et al., 2019). Moreover, it has been shown recently that inhibiting certain desaturase enzymes can protect against cell toxicity by reducing the formation of α-synuclein aggregates (Fanning et al., 2019). Despite emerging evidence of desaturases as potential pathological modulators, it is still not clear whether such cellular alterations caused due to disrupted lipid homeostasis have any effects on the overall PD pathophysiology including dopaminergic neuronal health.

*Caenorhabditis elegans* (*C. elegans*) offers several advantages for investigating both aging and neurodegenerative pathologies (Maulik et al., 2017). These nematode models have been used to characterize protein aggregation and dopaminergic neurodegeneration, key molecular hallmarks of PD (Cooper et al., 2015). In *C. elegans*, fat metabolism involves a complex and conserved gene network, which regulates food sensing and neuroendocrine signaling processes (Ashrafi et al., 2003). Food sensing behavior in worms is controlled by the dopaminergic system, which is also the crucial neural network affected in PD (Fu et al., 2014). *C. elegans* shares common human homologs of genes that are implicated in obesity and metabolics disorders (Ashrafi et al., 2003). One such enzyme called Δ9 desaturase is present in organisms from yeast to humans and is considered important for energy metabolism and lipid synthesis (Ntambi, 1999). Diets high in unsaturated fatty acids decrease expression of Δ9 desaturase and high carbohydrate consumption increases its expression (Brock et al., 2007). In *C. elegans*, Δ9 desaturases are encoded by the genes: *fat-5* and *fat-7*. RNA interference (RNAi) studies with *fat-5* and *fat-7* have shown that silencing of these genes reduces fat levels in the nematodes under normal physiological conditions (Van Gilst et al., 2005). On the contrary, *nhr-49* and *tub-1* which is a mammalian homolog of nuclear hormone receptors (NHRs), and tubby respectively, promotes fatty acid desaturation and beta-oxidation leading to high fat accumulation on gene silencing, and shortened lifespan in wild-type nematodes (Mukhopadhyay et al., 2005; Van Gilst et al., 2005; Pathare et al., 2012). Apart from that, fatty acid metabolism is known to be involved in stress resistance mechanisms and insulin signaling in the worm models (Horikawa and Sakamoto, 2009). Because of these previous interactions, the most well studied gene candidates of fatty acid metabolism: *fat-5, fat-7, nhr-49* and *tub-1* were targeted for the current study.

Based on this foundation, we chose to examine the effects of silencing fat metabolism genes in different worm models that recapitulate PD-like pathology. Using a genetic approach, we examined the effects of altered fat homeostasis on α-synuclein protein aggregation (wild-type and A53T mutation) and dopaminergic neuron degeneration. In addition, we also studied the overall effects on lifespan, healthspan and stress mechanisms. We hypothesized that silencing of *fat-5* and *fat-7* gene that codes for Δ9 desaturases will improve the pathophysiology and rescue several behavioral deficits, while silencing of *nhr-49* and *tub-1* will aggravate these endpoints in the worm models.

## Materials and Methods

### *C. elegans* Strains and Maintenance

Strains used for the study were OW13 (Punc54: α-syn; van Ham et al., 2008) and TG2435 (pDAT::GFP; Masoudi et al., 2014) which were acquired from the Caenorhabditis Genetics Center (University of Minnesota). The other strains for the study: JVR203 (pDAT::mut A53T-α-syn) and JVR208 (pDAT::WT-α-syn) were kindly donated by Drs. Shohei Mitani and Jeremy Van Raamsdonk. The OW13 strain expresses human wild-type: α-synuclein in its body wall muscle. The JVR208 and JVR203 strains express human wild-type and mutant A53T synuclein respectively in the dopaminergic neurons.

The animals were cultured on nematode growth medium (NGM) plates using standard procedure (Brenner, 1974) and maintained at 20°C. The plates were seeded with *Escherichia* *coli* strains OP-50-1 or HT115 depending on the experiments. Synchronous populations were created using the standard egg-lay method (allowing 20–30 animals to lay eggs for 4–6 h and then removing the adults) or by hypochlorite treatment (2% sodium hypochlorite in 0.5 M NaOH). All experimental plates (except for fecundity studies) contained 0.04 μg/ml fluorodeoxyuridine (FUdR) for progeny control.

### RNA Interference

RNAi was performed to assess the effect of silencing genes on the study end points. RNAi plates were prepared using isopropyl β-D-thiogalactoside (IPTG) and 4× concentrated HT115 bacteria for each gene target (*L4440, fat-5, fat-7, nhr-49* and *tub-1*) and covered with foil to protect from light (Scerbak et al., 2014; Maulik et al., 2018). For each replicate of the experiment (*n* = 10–15 animals/group per replicate), control experiments were performed to compare the mechanosensory touch function between *mec-7* silenced to empty vector (*L4440*) groups (O’Hagan et al., 2005), to ensure that the RNAi procedure worked properly (Supplementary Figure S1). The experimenter was blinded to the identity of the RNAi treatments. All RNAi clones were obtained from Open Biosystems (GE Dharmacon, Fisher Scientific).

### Measurement of Fat Content

Nile red staining was done to measure the fat content of the worms (Maulik et al., 2018). RNAi plates were seeded with HT115 bacteria (all five genetic treatments) mixed with nile red dye (Sigma) in the ratio of 1:500. Synchronous populations were created using the standard egg-lay method in NGM plates seeded with OP50-1 bacteria (allowing 20–30 animals to lay eggs for 4–6 h and then removing the adults). Following which, L3 larval animals were grown in RNAi + nile red plates until experimentation (*n* = 10–12 animals/group per replicate).

### Rotenone Administration

Rotenone (Sigma; 4 μM) in DMSO was added to bacteria-seeded NGM plates (Zhou et al., 2013). Larval L4 worms were then loaded onto the rotenone-coated plates until further experimentation. An equal volume of DMSO bacteria-seeded NGM plates were as vehicle controls.

### Lifespan Analysis

Following egg lay, synchronous populations of 44 L4 animals were transferred into each treatment plate for each replicate. The survival was determined daily by visual observation under dissecting microscope or by gentle prodding with a platinum wire. “Bagged” animals and animals crawling off the plates were censored (Scerbak et al., 2016). Each experiment was repeated at least three times.

### Body Bends

Body bends as a measure of healthspan were measured in Day 3 adult worms (*n* = 8–10 animals/group per replicate) by counting forward and complete sigmoidal movement of the nematodes for 1 min (Cooper et al., 2015). Spontaneous reversals were excluded.

### Fecundity

Fecundity or viable progeny count as a measure of healthspan was performed by plating Day 2 adults (peak fecundity) on fresh treatment plates (Hunter et al., 2018). The eggs laid by the adults were allowed to hatch and develop at 20°C for 48 h. The number of progeny produced was manually counted under a dissecting microscope (*n* = 8–10 animals/group per replicate).

### Pharyngeal Pump Rate

The pharyngeal pump rate as a measure of healthspan was assessed by manual counting in pharyngeal compressions for 30 s in Day 3 adult worms (*n* = 8–10 animals/group per replicate) by placing them on a seeded NGM assay plates (Hunter et al., 2018).

### Stress Resistance Assays: Heat Stress and Osmotic Stress

Sensitivity to heat stress was determined by assessing survival of young adult worms incubated at 35°C (*n* = 8–10 animals/group per replicate; Liu et al., 2015). Survival was assessed after 4 h. Sensitivity to osmotic stress was determined by transferring young adult worms to NGM plates containing 500 mM concentration of NaCl (*n* = 8–10 animals/group per replicate; Cooper et al., 2015). The survival of worms was determined after 24 h. Both stress assays were conducted on Day 3 of adulthood. The survival was determined by visual observation of motility under dissecting microscope or by gentle prodding with a platinum wire. The worms who failed to move on prodding were counted as dead.

### Dopamine Dependent Behaviors

#### Basal Response to Food

Day 3 worms were washed in M9 buffer and transferred to either unseeded NGM plates or NGM plates seeded with OP50-1 bacteria over the entire plate. After 3 min of acclimatization, the rate of movement was assessed by counting body bends for 30 s (Cooper et al., 2015). Basal slowing as a measure of dopaminergic function was calculated as the (rate of movement off food) − (rate of movement on food)/rate of movement off food (*n* = 10 animals/group per replicate).

#### Ethanol Avoidance Assay

Day 3 adult worms were transferred to the center of NGM plates divided into four quadrants: two quadrants seeded with 40 μl ethanol and two others contained no ethanol. After 30 min, the worms were scored for their presence in each quadrant. Ethanol avoidance as a measure of dopaminergic function was assessed as [(number of worms in control quadrants) − (number of worms in ethanol seeded quadrants)]/total number of worms (*n* = 10 animals/group per replicate; Cooper et al., 2015).

#### Microscopy and Image Quantification

All images of dopaminergic neuron degeneration were acquired with an Axiovert FX100 fluorescent microscope and all images of protein expression were acquired with a Zeiss LSM 510 laser scanning confocal microscope using z-stack function. Magnification and exposure settings were kept constant for each microscope. Images were analyzed with ImageJ (National Institute of Health). The α-synuclein levels were quantified based on the measurement of total fluorescence (Jadiya et al., 2011; Maulik et al., 2018). Dopaminergic neuron degeneration was quantified on the basis of scoring animals for the presence of normal and abnormal neurons. Any neuron with missing soma or dendrite, or with any morphological alterations like outgrowths or dendritic blebs were considered abnormal (Nass et al., 2002; Nass and Blakely, 2003). The data for neuronal degeneration was expressed as a percentage of normal neurons observed for each treatment group.

#### Statistical Analysis

The Kaplan-Meier log-rank analysis for lifespan survival experiments was performed with SPSS (Version 22, IBM, Inc.). All other statistical analyses were performed in Graph Pad Prism 8 (GraphPad Software, Inc.). Treatment effects among groups were assessed using one-way or two-way analysis of variance (ANOVA). Pairwise comparisons for to identify significant differences between treatment groups were tested using Tukey’s *post hoc* tests and *p*-values < 0.05 were considered significant (*p < 0.05, **p < 0.01, ***p < 0.001).

## Results

### Silencing of Fat Metabolism Genes Differentially Modulate Total Fat Content in Transgenic Models of *C. elegans*

**Figure 1:**
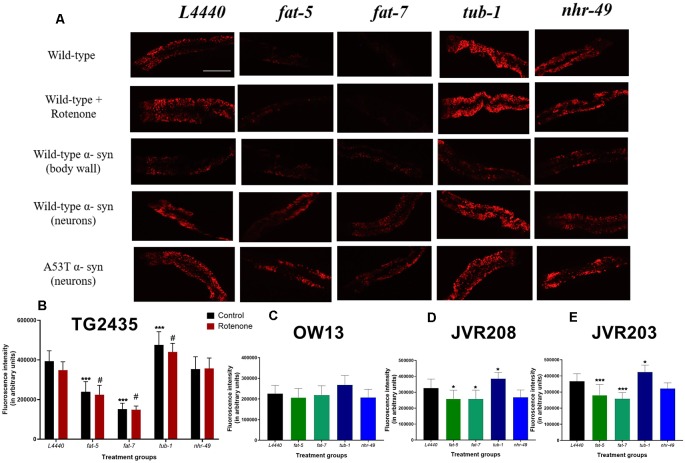
Silencing of fat metabolism genes resulted in differential fat levels in models of *Caenorhabditis elegans* *(C. elegans)*. **(A)** Confocal images and graphical representation of the Nile red staining as a measure of total fat content in **(B)** TG2435 **(C)** OW13 **(D)** JVR208 **(E)** JVR203 day 3 worms after gene silencing of fat metabolism genes. The worms (*n* = 10–12 per group) were grown on different RNA interference (RNAi) treatments: *L4440, fat-5, fat-7, nhr-49* and *tub-1* genes. Treatment with *fat-5* and *fat-7* genes (±4 μm rotenone) showed significantly low-fat content compared to the empty vector, *L4440* (^#^*p* < 0.05, ***p < 0.001 and *p < 0.05). Treatment with *tub*-1 (±4 μm rotenone) shows significantly high fat content than the *L4440* control (^#^*p* < 0.05, ***p < 0.001 and *p < 0.05). Each experiment was repeated two to three times. Scale bar 200 μm.

At day 3 of adulthood, genetic silencing of *fat-5* (*p* < 0.0001) and *fat-7* (*p* < 0.0001) genes in TG2435 model (dopaminergic neurons tagged with GFP) resulted in decreased fat content for both control and rotenone treated groups, compared to *L4440* groups (Figures 1A,B). There was significant increase in fat content on silencing *tub-1* gene for both control (*p* < 0.0001) and rotenone (*p* < 0.0001) groups, compared to *L4440*. However, there was no significant difference found for *nhr-49* group for either control or rotenone treatment (*p* > 0.5).

There were no significant differences found between any of the RNAi treatment groups for the OW13 model (wild-type α-synuclein expressed in body wall muscle) at day 3 of adulthood (*p* > 0.5, Figures 1A,C). Significant differences were found between RNAi treatment groups at day 3 of adulthood for model JVR208, in which human wild-type α-synuclein was expressed specifically in dopaminergic neurons (Figures 1A,D). Significant decrease in fat content was found for *fat-5* (*p* < 0.02) and *fat-7* (*p* < 0.02) groups, compared to the control: *L4440*. There were also elevated levels of fat found for *tub-1* (*p* < 0.05) group compared to the control. No significant effect was found for the *nhr-49* group (*p* > 0.5).

Significant effects were observed between the RNAi treatment groups for the JVR203 model (mutated A53T α-synuclein expressed in dopaminergic neurons) at day 3 of adulthood (Figures 1A,E). There was decrease in fat content observed for *fat-5* (*p* < 0.001) and *fat-7* (*p* < 0.001) groups, compared to the control: *L4440*. On the contrary, elevated levels of fat content were found for *tub-1* (*p* < 0.05) group compared to the control. There were no significant differences observed between *L4440* and *nhr-49* groups (*p* > 0.5).

### Silencing of *fat-5* and *fat-7* Genes Ameliorated α-Synuclein Expression and Improved Dopamine-Related Behaviors in Transgenic Models of *C. elegans*

**Figure 2:**
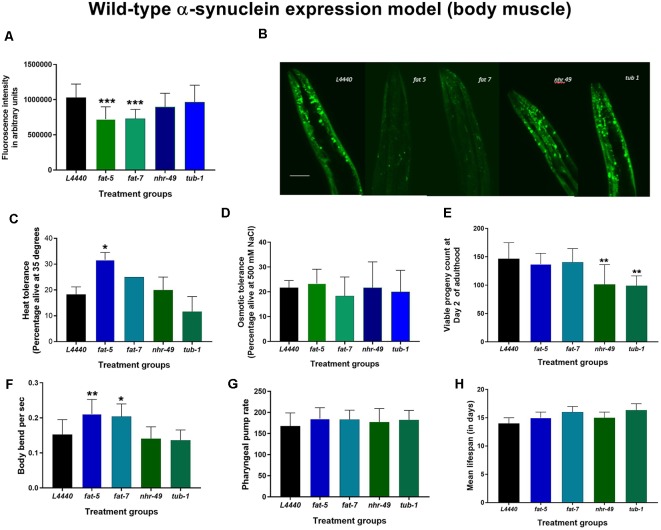
Silencing of *fat-5* and *fat-7* genes reduced human wild-type α-synuclein expression in OW13 model of *C. elegans*. Graphical representation of **(A)** fluorescence intensity of the OW13 (*n* = 20–30 animals per group). **(B)** Representative confocal images of the α-synuclein/YFP expression in the head region of day 7 OW13 adults, magnification 40× and scale bar 50 μm. **(C)** Heat tolerance and **(D)** osmotic tolerance **(E)** viable progeny **(F)** body bend **(G)** pharyngeal pump rate **(H)** lifespan of OW13 animals fed on different genetic RNAi treatments (*L4440, fat-5, fat-7, nhr-49* and *tub-1*). Empty vector L4440 was considered as control. The data represent the mean ± SEM with significant differences between the control and treatments at *p < 0.05, **p < 0.001 and ***p < 0.0001. Each experiment was repeated two to three times.

At day 7 of adulthood, silencing of *fat-5* and *fat-7* genes significantly reduced human wild-type α-synuclein expression in the OW13 model (Figures 2A,B) by about 30% when compared to the *L4440* control (*p* < 0.0001 and *p* < 0.0001, respectively). There were no significant differences among control, *nhr-49* and *tub-1* groups (*p* > 0.5).

**Figure 3:**
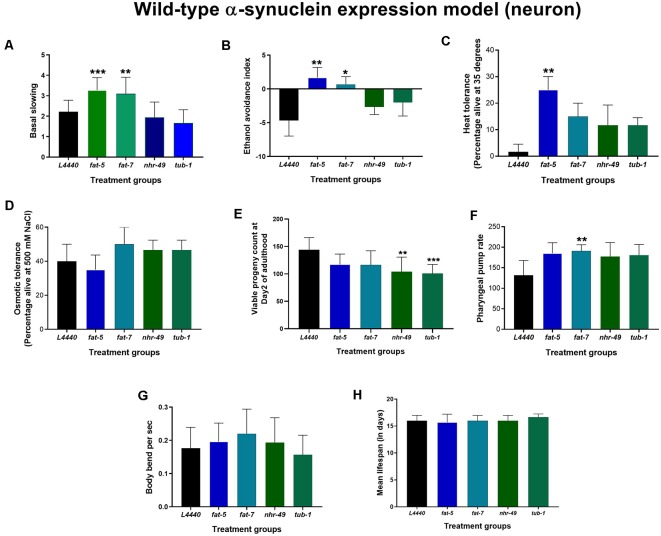
Genetic silencing of *fat-5* and *fat-7* genes improved dopamine-related behaviors in human wild-type α-synuclein expression JVR208 model of *C. elegans*. Graphical representation of **(A)** basal slowing **(B)** ethanol avoidance index **(C)** heat tolerance and **(D)** osmotic tolerance **(E)** viable progeny **(F)** pharyngeal pump rate **(G)** body bends (*n* = 10–15 animals per group; **H**) lifespan of JVR208 animals (*n* = 44 animals per group) fed on different genetic RNAi treatments (*L4440, fat-5, fat-7, nhr-49* and *tub-1*). Empty vector L4440 was considered as control. The data represent the mean ± SEM with significant differences between the control and treatments at *p < 0.05, **p < 0.01 and ***p < 0.001. Each experiment was repeated three times.

At day 3 of adulthood, dopaminergic functions were tested in another transgenic model (Figures 3A,B), JVR208, in which human wild-type α-synuclein was expressed specifically in dopaminergic neurons. Basal slowing was increased significantly for both *fat-5* and *fat-7* treated animals when compared to the *L4440* control group (*p* < 0.0008 and *p* < 0.007, respectively). Similarly, the ethanol avoidance index was significantly increased in animals in the *fat-5* and *fat-7* groups compared to the empty vector control (*p* < 0.008 and *p* < 0.03, respectively). There were no significant differences among control, *nhr-49* and *tub-1* groups (*p* > 0.5) for either of the behaviors.

### Genetic Silencing of *fat-5* and *fat-7* Genes Improved Resistance to Heat in Human Wild-Type α-Synuclein Expression Models of *C. elegans*

On RNAi treatment with the candidate fat metabolism genes in human wild-type α-synuclein expression models of *C. elegans* (both OW13 and JVR208), no significant effects (*p* > 0.5) were observed on the overall lifespan (Figures 2H, 3H; Supplementary Figures S2, S3) and osmotic tolerance (Figures 2D, 3D). Silencing the *nhr-49* (*p* < 0.003) and *tub-1* (*p* < 0.0009) genes significantly reduced viable number of progenies compared to the *L4440* control (Figures 2E, 3E). The pharyngeal pump rate was significantly increased in the *fat-7* (*p* < 0.04) group compared to the L4440 control group only for the JVR208 strain (Figure 3F). For the OW13 strain, there were no significant differences observed for pharyngeal pump rate between the treatment groups (Figure 2G; *p* > 0.05). The *fat-5* (*p* < 0.002) group showed significantly increased heat tolerance compared to the *L4440* group (Figures 2C, 3C) for both OW13 and JVR208 strains. Moreover, there was an increased number of body bends observed for the OW13 strain for both *fat-5* (*p* < 0.01) and *fat-7* (*p* < 0.05) groups (Figure 2F). No significant differences were found between the treatment groups for body bends in JVR208 strain (Figure 3G).

### Genetic Silencing of *fat-*5 and *fat-7* Genes Improved Dopaminergic Neuron Degeneration and Related Behaviors in a Model of *C. elegans* Expressing Human Mutated α-Synuclein (A53T Mutation)

**Figure 4:**
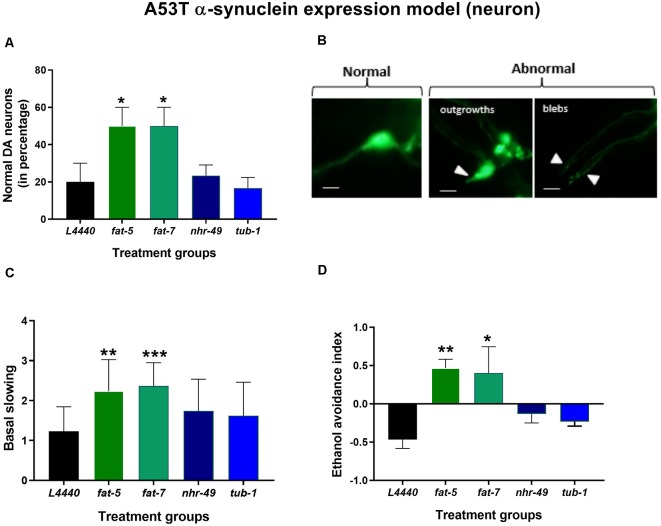
Silencing of *fat-5* and *fat-7* genes rescued dopaminergic degeneration and related behaviors in the model of *C. elegans* expressing human mutated α-synuclein (A53T mutation). Graphical representation of **(A)** percentage of normal dopaminergic neurons (*n* = 25–30 animals per group). **(B)** Representative images of dopaminergic normal and abnormal neurons with outgrowths and blebs, magnification 40× and scale bar 100 μm and **(C)** basal slowing and **(D)** ethanol avoidance behaviors (*n* = 10–15 animals) in JVR203 animals fed on different genetic RNAi treatments (*L4440, fat-5, fat-7, nhr-49* and *tub-1*). Empty vector L4440 was considered as control. The data represent the mean ± SEM with significant differences between the control and treatments at *p < 0.05, **p < 0.01 and ***p < 0.001. Each experiment was repeated three times.

Genetic silencing of *fat-5* (*p* < 0.02) and *fat-7* (*p* < 0.02) genes in a model of *C. elegans* expressing human mutated α-synuclein (A53T mutation) significantly increased the percentage of normal dopaminergic neuron morphology in *C. elegans* expressing human mutated α-synuclein in their dopaminergic neurons at day 7 of adulthood (Figures 4A,B) compared to the *L4440* control group. When compared to the *L4440* control group, both *fat-5* and *fat-7* groups showed significantly increased basal slowing behavior (Figure 4C; *p* < 0.003 and *p* < 0.0007, respectively) and ethanol avoidance index (Figure 4D; *p* < 0.0007 and *p* < 0.002, respectively). Silencing of the *nhr-49* and *tub-1* genes (Figure 4) had no significant effect on either dopaminergic neuron health (*p* > 0.5) nor its associated functional behaviors (*p* > 0.5).

### Deficits in Stress Resistance Was Improved by Silencing of *fat-*5 and *fat-7* Genes in *C. elegans* Expressing Human Mutated α-Synuclein (A53T mutation)

**Figure 5:**
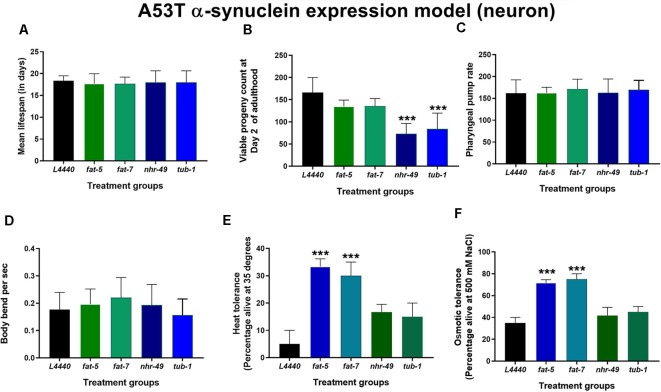
Defects in stress resistance are ameliorated by silencing of *fat-5* and *fat-7* genes in *C. elegans* expressing human mutated α-synuclein (A53T mutation). Graphical representation of **(A)** mean lifespan (*n* = 44 animals per group) **B**) viable progeny **(C)** pharyngeal pump rate **(D)** body bends **(E)** heat tolerance and **(F)** osmotic tolerance of JVR203 animals (*n* = 10–15 animals per group) fed on different genetic RNAi treatments (*L4440, fat-5, fat-7, nhr-49 and tub-1*). Empty vector L4440 was considered as control. The data represent the mean ± SEM with significant differences between the control and treatments at ***p < 0.001. Each experiment was repeated three times.

Silencing of the four fat genes in a model of *C. elegans* expressing human mutated α-synuclein (A53T mutation) had no significant impact (*p* > 0.5) on the overall lifespan (Figure 5A and Supplementary Figure S4), the pharyngeal pump rate (Figure 5C) and the number of body bends per second (Figure 5D). Silencing the *nhr-49* (*p* < 0.0002) and *tub-1* (*p* < 0.0002) genes significantly reduced the viable number of progenies compared to the *L4440* control group (Figure 5B). The heat tolerance was significantly increased in the *fat-5* (*p* < 0.002) and *fat-7* (*p* < 0.005) groups compared to the L4440 control group (Figure 5E). Silencing of the *fat-5* (*p* < 0.0002) and *fat-7* (*p* < 0.0002) genes also significantly increased osmotic tolerance compared to the L4440 control group (Figure 5F).

### Silencing of *fat-5* and *fat-7* Genes Rescued Dopaminergic Degeneration and Associated Behaviors in a Rotenone Induced Model of *C. elegans*

**Figure 6:**
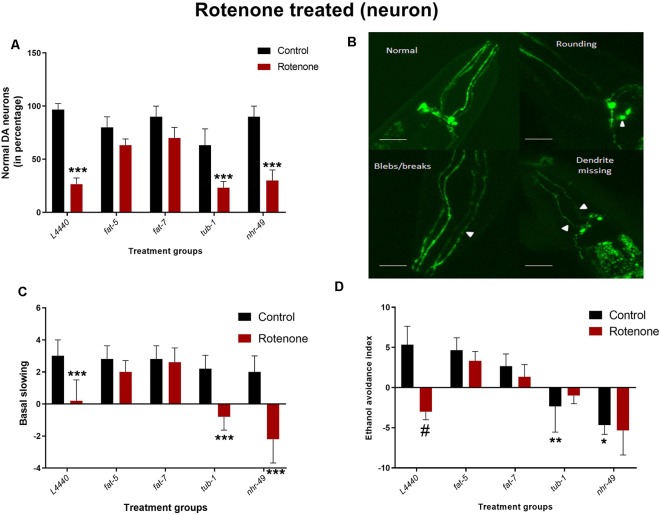
Dopaminergic degeneration and related behaviors are rescued by silencing *fat-5* and *fat-7* genes in a rotenone-induced model of *C. elegans*. Graphical representation of **(A)** percentage of normal dopaminergic neurons (*n* = 25–30 animals per group). **(B)** Representative images of dopaminergic neurons with rounding neurons or axons with blebs or missing dendrite, any neurons having either of these morphological features were considered as not normal, magnification 40× and scale bar 50 μm.**(C)** Basal slowing and **(D)** ethanol avoidance behaviors (*n* = 10–15 animals) in TG2435 animals fed on different genetic RNAi treatments (*L4440, fat-5, fat-7, nhr-49* and *tub-1*) and rotenone (0 and 4 μM). Empty vector L4440 was considered as control. The data represent the mean ± SEM with significant differences between the rotenone treated and untreated groups at ^#^*p* < 0.01, *p < 0.05, **p < 0.01 and ***p < 0.001. Each experiment was repeated three times.

At day 7 of adulthood (Figures 6A,B), *L4440* (*p* < 0.001), *nhr-49* (*p* < 0.02) and *tub-1* (*p* < 0.05) groups had significant neuronal degeneration after rotenone treatment compared to their respective vehicle control groups. However, there was no significant neuronal abnormality found for *fat-5* (*p* > 0.5) and *fat-7* (*p* > 0.5) groups treated with rotenone when compared to its respective control-treated groups.

Rotenone treatment significantly reduced basal slowing in *L4440* (*p* < 0.001), *nhr-49* (*p* < 0.001) and *tub-1* (*p* < 0.002) group (Figure 6C). However, exposing the nematodes to rotenone did not have any negative effect on *fat-5* (*p* > 0.5) and *fat-7* (*p* > 0.5) groups compared to their vehicle control groups.

Animals treated with rotenone in the *L4440* (*p* < 0.0003) groups showed significant reductions in the ethanol avoidance index compared to their vehicle control groups (Figure 6D). However, there were no significant differences between rotenone and vehicle control groups for any other genetic treatments. Moreover, the total scores in the vehicle-treated *nhr-49* (*p* < 0.002) and *tub-1* (*p* < 0.02) groups was significantly lower than the *L4440* group.

### Lifespan or Healthspan Measures Were Unaltered by Silencing of Fat Genes in Rotenone Induced Model of *C. elegans*

**Figure 7:**
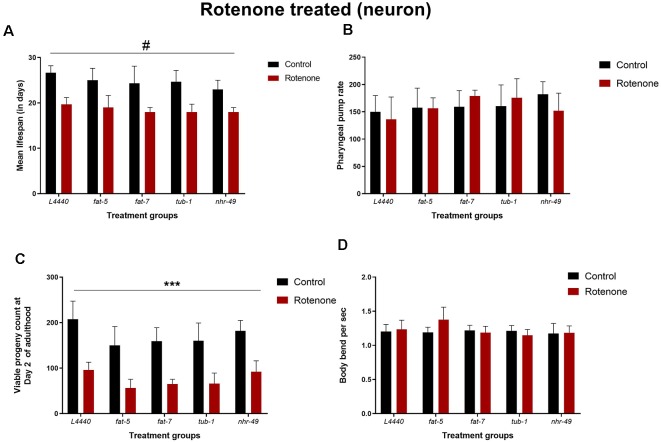
Lifespan or healthspan measures are unaltered by silencing of *fat-5* and *fat-7* genes in rotenone-induced model of *C. elegans*. Graphical representation of **(A)** mean lifespan (*n* = 44 animals per group; **B**) pharyngeal pump rate **(C)** viable progeny and **(D)** body bends of TG2435 animals (*n* = 8–10 animals per group) fed on different genetic RNAi treatments (*L4440, fat-5, fat-7, nhr-49* and *tub-1*) and rotenone (0 and 4 μM). Empty vector L4440 was considered as control. The data represent the mean ± SEM with significant differences between the control and rotenone treatments at ^#^*p* < 0.01 and ***p < 0.001. Each experiment was repeated three times.

After rotenone treatment, the lifespan of all the RNAi groups was significantly reduced compared to the untreated controls (*p* < 0.05), without significant differences among the RNAi groups (*p* > 0.5; Figure 7A and Supplementary Figure S5). Similarly, rotenone administration reduced the total progeny produced for all the RNAi groups compared to the vehicle control groups (*p* < 0.0001), without significant differences among the RNAi groups (*p* > 0.5; Figure 7C). There was no significant effect for the pharyngeal pump rate (Figure 7B; *p* > 0.1) and the number of body bends per second (Figure 7D; *p* > 0.1).

## Discussion

Neurodegenerative disorders like PD contributes to pathological changes in the brain such as atrophy of neurons and cellular overload (Hindle, 2010). Fat metabolism plays an important role in cell physiology such as energy metabolism and signal transduction processes (Ashrafi et al., 2003). Since PD is often characterized by dysfunction of cell homeostasis and energy metabolism (Adibhatla and Hatcher, 2008), the role of fat signaling in influencing the disease pathophysiology such as protein aggregation and neuronal degeneration cannot be ruled out. Though there has been extensive research on the molecular mechanisms leading to cell death in PD pathology, the role of fat metabolism and more specifically Δ9 desaturase in influencing such molecular hallmarks remains unclear. In *C. elegans*, aging is represented by healthspan measures which is analogous to humans (Tissenbaum, 2012). These measures include reduced pharyngeal pump rate, decline in motility and fertility and changes in neuromuscular function (Uno and Nishida, 2016). Moreover, environmental stress response such as heat shock and osmotic stress has been used in nematodes to unravel genetic machineries of many diseases (Rodriguez et al., 2013). The transgenic PD-like models of *C. elegans* have shown to possess deficits in the form of reduced lifespan, healthspan and increased sensitivity to environmental stressors (Cooper et al., 2015). These parameters were also used as indicators to characterize age-related behavioral measures in our study. Through genetic manipulations, we demonstrate how silencing of Δ9 desaturase encoding *fat-*5 and *fat-7* in worms can improve key features of PD: protein aggregation, neuronal degeneration and dopamine-dependent behaviors (Supplementary Table S1).

Like others (Zhou et al., 2013; Cooper et al., 2015), our experiments show how proteotoxic stress (in the form of α-synuclein expression) or rotenone-induced neuronal degeneration in *C. elegans* can modify dopaminergic related functions or healthspan (Figures 2, 4, 6). Moreover, toxic protein (α-synuclein) expression in their neurons can result in reduced basal levels of heat and osmotic stress tolerance. Most strikingly, our study highlights that genetic manipulation of desaturase encoding *fat-5* and *fat-7* genes improves the pathophysiology in the PD-like worm strains (α-synuclein and rotenone-induced model). Silencing these genes reduced α-synuclein expression and protected against neuronal damage (Figures 2, 4, 6). Additionally, it also improved dopamine behaviors, including basal slowing and ethanol avoidance in the nematodes (Figures 2, 4, 6). One of the probable mechanisms through which the desaturase enzyme influences α-synuclein toxicity could be linked with membrane vesicular trafficking (Vincent et al., 2018). α-synuclein is mostly speculated to interact with the membrane lipids and disrupt vesicular trafficking, which is a key element for the functioning of neurotransmitters like dopamine (Alter et al., 2013; Vincent et al., 2018). The genes mediating vesicular trafficking and α-synuclein toxicity have been studied in different organisms (Khurana et al., 2017). Recently, two studies also have highlighted the role of desaturases in ameliorating PD-like pathology in different model systems (Fanning et al., 2019; Vincent et al., 2018). In congruence, our study has focused on how components of aging (like healthspan, lifespan and stress tolerance) can be intertwined with the neurodegenerative pathology. We also demonstrate the role of Δ9 desaturase in models where PD-like pathology is inflicted due to environmental toxins (rotenone). Our results, therefore, provide a comprehensive evaluation of the differential effects of contributing factors (genetic and environmental) in modulating PD-like pathology due to altered fat homeostasis. Most intriguingly, our study shows that RNAi of *fat-5*, *fat-7* and *tub-1* resulted in differential levels of fat depending on the promoter (body muscle vs. neurons) used to express the α-synuclein protein (Figure 1). It was interesting to note that genetic silencing of the selected fat metabolism genes had no effect on the total fat levels of OW13 strain which already demonstrated low basal fat profile due to α-synuclein expression in the body muscle (Maulik et al., 2018; Figure 1C). This could be largely due to other compensatory transcriptional machinery that aid in maintaining fat homeostasis (Ashrafi, 2007) and are unique to degenerative conditions. To our surprise, rotenone treatment that alters dopaminergic neuronal structure and function did not alter the fat content in the nematodes on its own (Figure 1). This suggests that changes in total cellular fat content may not be a direct correlation of genetic manipulation and such alterations can depend on the extrinsic factors such as food availability (Brock et al., 2007). Though studies in both *C. elegans* and other models have shown the importance of dopamine signaling in modulating fat metabolism (Wang et al., 2001; Palmiter, 2007; Barros et al., 2014), such complicated interactions still remain elusive and raise further questions. Considering the fact that altered lipid levels are integral to PD pathophysiology (Fabelo et al., 2011), it is also possible that genetic knockdown of *fat-5* and *fat-7* genes may result in changes in lipid profile or partitioning (rather than total lipid content) which in turn can modulate neuronal health by other signaling mechanisms. Interestingly, such associations between fat metabolism and these degenerative molecular pathologies in worm models have not been previously explored. This exploration of the complex interaction between fat metabolism genes and physiological assessments of PD-like pathology, therefore, warrants further investigation.

Research shows that stress-resistance mechanisms are controlled by fatty acid metabolism (Horikawa and Sakamoto, 2009). Moreover, PD-like models can have differential sensitivity towards various stressors (Cooper et al., 2015). Like others, our research showed that strains with proteotoxic stress can have a very low tolerance to heat or osmotic stress (Figures 3C,D, 5E,F). We also demonstrated that RNAi of *fat-5* and *fat-7* increased heat tolerance in protein expression (either wild-type/A53T α-synuclein/or both) models of *C. elegans* probably due to reduced unsaturated fats which are known to impact thermotolerance (Horikawa and Sakamoto, 2009). Similarly, silencing of *fat-5* and *fat-7* improved osmotic tolerance in A53T α-synuclein model but not in the wild-type α-synuclein strain. Apart from strain differences, these observed disparate effects might also be attributed to varying changes in the mitochondrial proteome and function due to the expression of functional (wild-type) vs. mutated protein (Pennington et al., 2010). Furthermore, mitochondrial function is often considered crucial for both heat and osmotic tolerance (Pastor et al., 2009; Li et al., 2019).

Silencing of the *nhr-49* and *tub-1* genes significantly reduced ethanol avoidance behavior only in the wild-type strain, TG2435 (Figures 6C,D). In mammals, PPARα, a *nhr-49* homolog, is known to negatively modulate dopamine cell activity (Melis et al., 2010). Moreover, ethanol avoidance in worms is regulated by both dopamine and serotonin and such an environmental response is co-dependent on olfactory neurons (Lee et al., 2009). Our results also show that silencing only *tub-1 (*and not *nhr-49)* increases fat levels in PD worm models. Previous study indicates that modulation of fat content due to silencing of *nhr-49* can actually depend on the age of the worms (Van Gilst et al., 2005). Genetic manipulation of both these genes (*tub-1* and *nhr-49*) led to reduced fertility in proteotoxic worm models of PD (Van Gilst et al., 2005). However, silencing of either of these genes had no effect on α-synuclein protein expression or dopaminergic functions (in both wild-type and A53T strains). The lipid metabolism in *C. elegans* is well deciphered and less complex than the higher mammalian systems, indicating further scrutiny of other genetic components and compensatory mechanisms that might have led to such differential effects.

Finally, results from our research reveals that silencing of *fat-5* and *fat-7* genes may influence neuronal health or protein expression greatly, though its effect on lifespan and general health can be marginal or none. Cooper et al. (2015) have primarily used lifespan as the key parameter for measuring anti-aging interventions. Previous research has clearly shown that lifespan and healthspan can be considered as standalone events in organisms and increased lifespan often is accompanied by frail health or trade-offs like reduced fertility (Bansal et al., 2015; Scerbak et al., 2016). Therefore, factors controlling lifespan and healthspan could be divergent events (Apfeld and Fontana, 2017). This could explain our differences in results where none of the fat genes had any impact on the longevity of the PD models. Hence, based on our findings we may conclude that modulating lifespan should not be the sole focus for anti-aging treatments especially for therapeutic options of age-related disorders like PD. In light of our findings, it should be noted that *C. elegans* is a simple *in vivo* model system compared to the actual human pathology which is intricate and results due to a complex interaction of various factors such as, sex, age, environment and genetics. Also, lack of an endogenous α-synuclein and therefore absence of formation of protein aggregates due to rotenone administration may add up to the limitation of the model organism itself. Nevertheless, studies as ours provide a foundation for understanding how genetic and environmental perturbation can alter cellular functionalities by dysregulating proteins in their native and mutated state, ultimately leading to cellular anomaly. Understanding these interactions in a simpler model system can be paralleled in higher organisms for unraveling complex mechanisms. Our findings, therefore, form the basis for new therapeutic targets in human PD patients and screen important homologous lipid genes encoding for desaturases and tubby with an aim to further connect metabolism and nervous system disorders.

## Conclusion

Overall, our results screen fat metabolism genes and show that perturbing fat homeostasis through genetic silencing of Δ9 desaturases can attenuate PD pathological hallmarks by reducing toxic protein expression and rescuing neuronal loss in the nematode models. Furthermore, it also poses questions on the intricate interplay between fat metabolism pathways and neurodegenerative disorders.

## Author Contributions

MM and SM conducted all the experiments, led concept generation, data interpretation, manuscript writing efforts and statistical analysis. AB and BL supported study set-up and conducted all experiments. BT contributed to manuscript review. AB-I made significant contributions to research design, data interpretation and manuscript preparation.

## Conflict of Interest Statement

The authors declare that the research was conducted in the absence of any commercial or financial relationships that could be construed as a potential conflict of interest.

## Supplementary Material

FIGURE S1 Graphical representation of the touch response as a measure of batch control for the RNAi plates. The worms (*n* = 10 per group) were grown on two different RNAi treatments: *L4440* and *mec-7*. Treatment with *mec-7* showed significantly diminished mechanosensory touch response compared to the L4440 control (****p* < 0.001).

FIGURE S2 Representative Kaplan-Meir survival curve for wild-type α-synuclein overexpression strain (OW13) treated with RNAi treatments: *L4440, fat-5, fat-7, nhr-49* and *tub-1*. There was no significant difference in lifespan extension (*p* > 0.05; Kaplan-Meier log-rank test) between the groups. Each experiment was repeated at least twice (*n* = 44 animals per treatment group).

FIGURE S3 Representative Kaplan-Meir survival curve for wild-type α-synuclein overexpression strain (JVR208) treated with RNAi treatments: *L4440, fat-5, fat-7, nhr-49* and *tub-1*. There was no significant difference in lifespan extension (*p* > 0.05; Kaplan-Meier log-rank test) between the groups. Each experiment was repeated at least thrice (*n* = 44 animals per treatment group).

FIGURE S4 Representative Kaplan-Meir survival curve for mutant A53T α-synuclein overexpression strain (JVR203) treated with RNAi treatments: *L4440, fat-5, fat-7, nhr-49* and *tub-1*. There was no significant difference in lifespan extension (*p* > 0.05; Kaplan-Meier log-rank test) between the groups. Each experiment was repeated at least thrice (*n* = 44 animals per treatment group).

FIGURE S5 Representative Kaplan-Meir survival curve for wild-type strain (TG2435) treated with RNAi treatments : *L4440, fat-5, fat-7, nhr-49* and *tub-1* and with rotenone (0 and 4 μM). There was significant difference in lifespan (*p* < 0.05; Kaplan-Meier log-rank test) between the rotenone-treated groups. Each experiment was repeated at least thrice (*n* = 44 animals per treatment group).

TABLE S1 Summary of the effect of silencing of fat metabolism genes on tested outcomes in PD models of *C. elegans*.

## References

[B1] Adibhatla R. M. Hatcher J. F. (2008). Altered lipid metabolism in brain injury and disorders. Subcell. Biochem. 49, 241–268. 10.1007/978-1-4020-8831-5_9 18751914 PMC2293298

[B2] Alter S. P. Lenzi G. M. Bernstein A. I. Miller G. W. (2013). Vesicular integrity in Parkinson’s disease. Curr. Neurol. Neurosci. Rep. 13:362. 10.1007/s11910-013-0362-3 23690026 PMC4019229

[B3] Apfeld J. Fontana W. (2017). Age-dependence and aging-dependence: neuronal loss and lifespan in a *C. elegans* model of Parkinson’s disease. Biology 7:1. 10.3390/biology7010001 29295479 PMC5872027

[B5] Ashrafi K. (2007). Obesity and the regulation of fat metabolism. WormBook 9, 1–20. 10.1895/wormbook.1.130.1 PMC4780880 18050496

[B4] Ashrafi K. Chang F. Y. Watts J. L. Fraser A. G. Kamath R. S. Ahringer J.. (2003). Genome-wide RNAi analysis of *Caenorhabditis elegans* fat regulatory genes. Nature 421, 268–272. 10.1038/nature01279 12529643

[B6] Bansal A. Zhu L. J. Yen K. Tissenbaum H. A. (2015). Uncoupling lifespan and healthspan in *Caenorhabditis elegans* longevity mutants. Proc. Natl. Acad. Sci. U S A 112, E277–E286. 10.1073/pnas.1412192112 25561524 PMC4311797

[B7] Barber C. N. Raben D. M. (2019). Lipid metabolism crosstalk in the brain: glia and neurons. Front. Cell. Neurosci. 13:212. 10.3389/fncel.2019.00212 31164804 PMC6536584

[B8] Barros A. G. Bridi J. C. de Souza B. R. de Castro Júnior C. de Lima Torres K. C. Malard L.. (2014). Dopamine signaling regulates fat content through β-oxidation in *Caenorhabditis elegans*. PLoS One 9:e85874. 10.1371/journal.pone.0085874 24465759 PMC3899111

[B9] Beitz J. M. (2014). Parkinson’s disease: a review. Front. Biosci. 6, 65–74. 10.2741/S415 24389262

[B10] Brenner S. (1974). The genetics of *Caenorhabditis elegans*. Genetics 77, 71–94. 4366476 10.1093/genetics/77.1.71 PMC1213120

[B11] Brock T. J. Browse J. Watts J. L. (2007). Fatty acid desaturation and the regulation of adiposity in *Caenorhabditis elegans*. Genetics 176, 865–875. 10.1534/genetics.107.071860 17435249 PMC1894614

[B12] Cooper J. F. Dues D. J. Spielbauer K. K. Machiela E. Senchuk M. M. Van Raamsdonk J. M. (2015). Delaying aging is neuroprotective in Parkinson’s disease: a genetic analysis in, *C. elegans* models. NPJ Parkinsons Dis. 1:15022. 10.1038/npjparkd.2015.22 28725688 PMC5516561

[B13] Elbaz A. Tranchant C. (2007). Epidemiologic studies of environmental exposures in Parkinson’s disease. J. Neurol. Sci. 262, 37–44. 10.1016/j.jns.2007.06.024 17673256

[B15] Fabelo N. Martín V. Santpere G. Marín R. Torrent L. Ferrer I.. (2011). Severe alterations in lipid composition of frontal cortex lipid rafts from Parkinson’s disease and incidental Parkinson’s disease. Mol. Med. 17, 1107–1118. 10.2119/molmed.2011.00119 21717034 PMC3188884

[B16] Fanning S. Haque A. Imberdis T. Baru V. Barrasa M. I. Nuber S.. (2019). Lipidomic analysis of α-synuclein neurotoxicity identifies stearoyl CoA desaturase as a target for Parkinson treatment. Mol. Cell 73, 1001.e8–1014.e8. 10.1016/j.molcel.2018.11.028 30527540 PMC6408259

[B17] Fu R.-H. Harn H.-J. Liu S.-P. Chen C.-S. Chang W.-L. Chen Y.-M.. (2014). n-butylidenephthalide protects against dopaminergic neuron degeneration and α-synuclein accumulation in *Caenorhabditis elegans* models of Parkinson’s disease. PLoS One 9:e85305. 10.1371/journal.pone.0085305 24416384 PMC3885701

[B18] Goldman S. M. Quinlan P. J. Ross G. W. Marras C. Meng C. Bhudhikanok G. S.. (2012). Solvent exposures and Parkinson disease risk in twins. Ann. Neurol. 71, 776–784. 10.1002/ana.22629 22083847 PMC3366287

[B20] Hindle J. V. (2010). Ageing, neurodegeneration and Parkinson’s disease. Age Ageing 39, 156–161. 10.1093/ageing/afp223 20051606

[B21] Horikawa M. Sakamoto K. (2009). Fatty-acid metabolism is involved in stress-resistance mechanisms of *Caenorhabditis elegans*. Biochem. Biophys. Res. Commun. 390, 1402–1407. 10.1016/j.bbrc.2009.11.006 19896458

[B22] Hunter S. Maulik M. Scerbak C. Vayndorf E. Taylor B. E. (2018). *Caenorhabditis* sieve: a low-tech instrument and methodology for sorting small multicellular organisms. J. Vis. Exp. 4:e58014. 10.3791/58014 30035770 PMC6124601

[B23] Jadiya P. Khan A. Sammi S. R. Kaur S. Mir S. S. Nazir A. (2011). Anti-Parkinsonian effects of Bacopa monnieri: insights from transgenic and pharmacological *Caenorhabditis elegans* models of Parkinson’s disease. Biochem. Biophys. Res. Commun. 413, 605–610. 10.1016/j.bbrc.2011.09.010 21925152

[B24] Khurana V. Peng J. Chung C. Y. Auluck P. K. Fanning S. Tardiff D. F.. (2017). Genome-scale networks link neurodegenerative disease genes to α-synuclein through specific molecular pathways. Cell Syst. 4, 157.e14–170.e14. 10.1016/j.cels.2016.12.011 28131822 PMC5388136

[B25] Lee J. Jee C. McIntire S. L. (2009). Ethanol preference in, *C. elegans*. Genes Brain Behav. 8, 578–585. 10.1111/j.1601-183x.2009.00513.x 19614755 PMC2880621

[B26] Li X. C. Peris D. Hittinger C. T. Sia E. A. Fay J. C. (2019). Mitochondria-encoded genes contribute to evolution of heat and cold tolerance in yeast. Sci. Adv. 5:eaav1848. 10.1126/sciadv.aav1848 30729162 PMC6353624

[B27] Liu J. Banskota A. H. Critchley A. T. Hafting J. Prithiviraj B. (2015). Neuroprotective effects of the cultivated chondrus crispus in a *C. elegans* model of Parkinson’s disease. Mar. Drugs 13, 2250–2266. 10.3390/md13042250 25874922 PMC4413210

[B28] Masoudi N. Ibanez-Cruceyra P. Offenburger S. L. Holmes A. Gartner A. (2014). Tetraspanin (TSP-17) protects dopaminergic neurons against 6-OHDA-induced neurodegeneration in *C. elegans*. PLoS Genet. 10:e1004767. 10.1371/journal.pgen.1004767 25474638 PMC4256090

[B29] Maulik M. Mitra S. Bult-Ito A. Taylor B. E. Vayndorf E. M. (2017). Behavioral phenotyping and pathological indicators of Parkinson’s disease in *C. elegans* models. Front. Genet. 8:77. 10.3389/fgene.2017.00077 28659967 PMC5468440

[B30] Maulik M. Mitra S. Hunter S. Hunstiger M. Oliver S. R. Bult-Ito A.. (2018). Sir-2.1 mediated attenuation of alpha-synuclein expression by Alaskan bog blueberry polyphenols in a transgenic model of *Caenorhabditis elegans*. Sci. Rep. 8:10216. 10.1038/s41598-018-26905-4 29976995 PMC6033853

[B31] Maulik M. Mitra S. Sweeney M. Lu B. Taylor B. E. Bult-Ito A. (2019). Complex interaction of dietary fat and Alaskan bog blueberry supplementation influences manganese mediated neurotoxicity and behavioral impairments. J. Funct. Foods 53, 306–317. 10.1016/j.jff.2018.12.028 PMC6761827 31558914

[B32] Melis M. Carta S. Fattore L. Tolu S. Yasar S. Goldberg S. R.. (2010). Peroxisome proliferator-activated receptors-alpha modulate dopamine cell activity through nicotinic receptors. Biol. Psychiatry 68, 256–264. 10.1016/j.biopsych.2010.04.016 20570248 PMC2907468

[B33] Michel P. P. Toulorge D. Guerreiro S. Hirsch E. C. (2013). Specific needs of dopamine neurons for stimulation in order to survive: implication for Parkinson disease. FASEB J. 27, 3414–3423. 10.1096/fj.12-220418 23699175

[B34] Mukhopadhyay A. Deplancke B. Walhout A. J. Tissenbaum H. A. (2005). *C. elegans* tubby regulates life span and fat storage by two independent mechanisms. Cell Metab. 2, 35–42. 10.1016/j.cmet.2005.06.004 16054097

[B36] Nass R. Blakely R. D. (2003). The *Caenorhabditis elegans* dopaminergic system: opportunities for insights into dopamine transport and neurodegeneration. Annu. Rev. Pharmacol. Toxicol. 43, 521–544. 10.1146/annurev.pharmtox.43.100901.135934 12415122

[B37] Nass R. Hall D. H. Miller D. M. Blakely R. D. (2002). Neurotoxin-induced degeneration of dopamine neurons in *Caenorhabditis elegans*. Proc. Natl. Acad. Sci. U S A 99, 3264–3269. 10.1073/pnas.042497999 11867711 PMC122507

[B38] Ntambi J. M. (1999). Regulation of stearoyl-CoA desaturase by polyunsaturated fatty acids and cholesterol. J. Lipid Res. 40, 1549–1558. 10484602

[B39] O’Hagan R. Chalfie M. Goodman M. B. (2005). The MEC-4 DEG/ENaC channel of *Caenorhabditis elegans* touch receptor neurons transduces mechanical signals. Nat. Neurosci. 8, 43–50. 10.1038/nn1362 15580270

[B40] Palmiter R. D. (2007). Is dopamine a physiologically relevant mediator of feeding behavior? Trends Neurosci. 30, 375–381. 10.1016/j.tins.2007.06.004 17604133

[B41] Pastor M. M. Proft M. Pascual-Ahuir A. (2009). Mitochondrial function is an inducible determinant of osmotic stress adaptation in yeast. J. Biol. Chem. 284, 30307–30317. 10.1074/jbc.M109.050682 19720830 PMC2781586

[B42] Pathare P. P. Lin A. Bornfeldt K. E. Taubert S. Van Gilst M. R. (2012). Coordinate regulation of lipid metabolism by novel nuclear receptor partnerships. PLoS Genet. 8:e1002645. 10.1371/journal.pgen.1002645 22511885 PMC3325191

[B43] Pennington K. Peng J. Hung C. C. Banks R. E. Robinson P. A. (2010). Differential effects of wild-type and A53T mutant isoform of alpha-synuclein on the mitochondrial proteome of differentiated SH-SY5Y cells. J. Proteome Res. 9, 2390–2401. 10.1021/pr901102d 20334438

[B44] Polymeropoulos M. H. Lavedan C. Leroy E. Ide S. E. Dehejia A. Dutra A.. (1997). Mutation in the alpha-synuclein gene identified in families with Parkinson’s disease. Science 276, 2045–2047. 10.1126/science.276.5321.2045 9197268

[B45] Rodriguez M. Snoek L. B. De Bono M. Kammenga J. E. (2013). Worms under stress: *C. elegans* stress response and its relevance to complex human disease and aging. Trends Genet. 29, 367–374. 10.1016/j.tig.2013.01.010 23428113

[B46] Scerbak C. Vayndorf E. M. Hernandez A. McGill C. Taylor B. E. (2016). Mechanosensory neuron aging: differential trajectories with lifespan-extending alaskan berry and fungal treatments in *Caenorhabditis elegans*. Front. Aging Neurosci. 8:173. 10.3389/fnagi.2016.00173 27486399 PMC4947587

[B47] Scerbak C. Vayndorf E. M. Parker J. A. Neri C. Driscoll M. Taylor B. E. (2014). Insulin signaling in the aging of healthy and proteotoxically stressed mechanosensory neurons. Front. Genet. 5:212. 10.3389/fgene.2014.00212 25101108 PMC4107846

[B48] Shulman J. M. De Jager P. L. Feany M. B. (2011). Parkinson’s disease: genetics and pathogenesis. Annu. Rev. Pathol. 6, 193–222. 10.1146/annurev-pathol-011110-130242 21034221

[B49] Spillantini M. G. (1999). Parkinson’s disease, dementia with lewy bodies and multiple system atrophy are alpha-synucleinopathies. Parkinsonism Relat. Disord. 5, 157–162. 10.1016/s1353-8020(99)00031-0 18591134

[B50] Tissenbaum H. A. (2012). Genetics, life span, health span and the aging process in *Caenorhabditis elegans*. J. Gerontol. A Biol. Sci. Med. Sci. 67, 503–510. 10.1093/gerona/gls088 22499764 PMC3410663

[B51] Uno M. Nishida E. (2016). Lifespan-regulating genes in, *C. elegans*. NPJ Aging Mech. Dis. 2:16010. 10.1038/npjamd.2016.10 28721266 PMC5514992

[B52] Van Gilst M. R. Hadjivassiliou H. Jolly A. Yamamoto K. R. (2005). Nuclear hormone receptor NHR-49 controls fat consumption and fatty acid composition in, *C. elegans*. PLoS Biol. 3:e53. 10.1371/journal.pbio.0030053 15719061 PMC547972

[B53] van Ham T. J. Thijssen K. L. Breitling R. Hofstra R. M. W. Plasterk R. H. A. Nollen E. A. A. (2008). *C. elegans* model identifies genetic modifiers of alpha-synuclein inclusion formation during aging. PLoS Genet. 4:e1000027. 10.1371/journal.pgen.1000027 18369446 PMC2265412

[B54] Vincent B. M. Tardiff D. F. Piotrowski J. S. Aron R. Lucas M. C. Chung C. Y.. (2018). Inhibiting stearoyl-CoA desaturase ameliorates α-synuclein cytotoxicity. Cell Reports 25, 2742.e31–2754.e31. 10.1016/j.celrep.2018.11.028 30517862

[B55] Wang G. J. Volkow N. D. Logan J. Pappas N. R. Wong C. T. Zhu W.. (2001). Brain dopamine and obesity. Lancet 357, 354–357. 10.1016/s0140-6736(00)03643-6 11210998

[B56] Zhou S. Wang Z. Klaunig J. E. (2013). *Caenorhabditis elegans* neuron degeneration and mitochondrial suppression caused by selected environmental chemicals. Int. J. Biochem. Mol. Biol. 4, 191–200. Available online at: http://www.ijbmb.org/files/ijbmb1309002.pdf. 24380023 PMC3867705

