# Supplementary material for: Genetic Silencing of Fatty Acid Desaturases Modulates α-Synuclein Toxicity and Neuronal Loss in Parkinson-Like Models of C. elegans
Source: Front Aging Neurosci. 2019 Aug 6;11:207. doi: 10.3389/fnagi.2019.00207 (PMC6691153; doi:10.3389/fnagi.2019.00207)
Supplement: FIGURE S1 — Graphical representation of the touch response as a measure of batch control for the RNAi plates. The worms (n = 10 per group) were grown on two different RNAi treatments: L4440 and mec-7. Treatment with mec-7 showed significantly diminished mechanosensory touch response compared to the L4440 control (***p < 0.001). [file Data_Sheet_1.doc]

**Title**: Genetic silencing of fatty acid desaturases modulates α-synuclein toxicity and neuronal loss in Parkinson-like models of *C. elegans*

Supplemental Figure 1 Graphical representation of the touch response as a measure of batch control for the RNAi plates.

The worms (n=10 per group) were grown on two different RNAi treatments: *L4440* and *mec-7.*  Treatment with *mec-7* showed significantly diminished mechanosensory touch response compared to the L4440 control (***p<0.001).


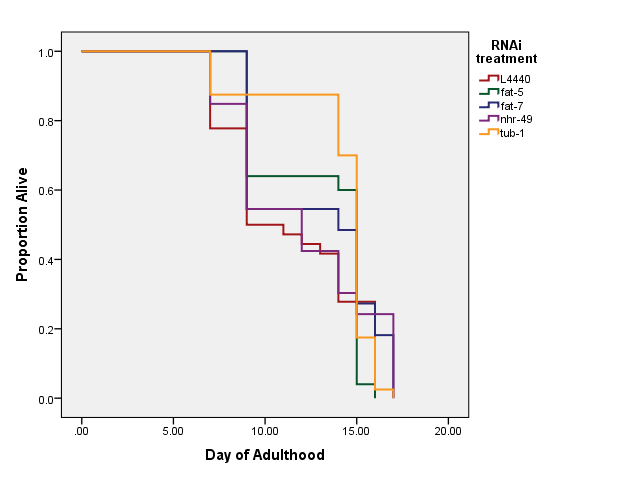


Supplemental Figure 2. Representative Kaplan-Meir survival curve for wild-type alpha-synuclein overexpression strain (OW13) treated with RNAi treatments : *L4440, fat-5, fat-7, nhr-49 and tub-1* . There was no significant difference in lifespan extension (p > 0.05; Kaplan–Meier log-rank test) between the groups . Each experiment was repeated at least twice (n=44 animals per treatment group).


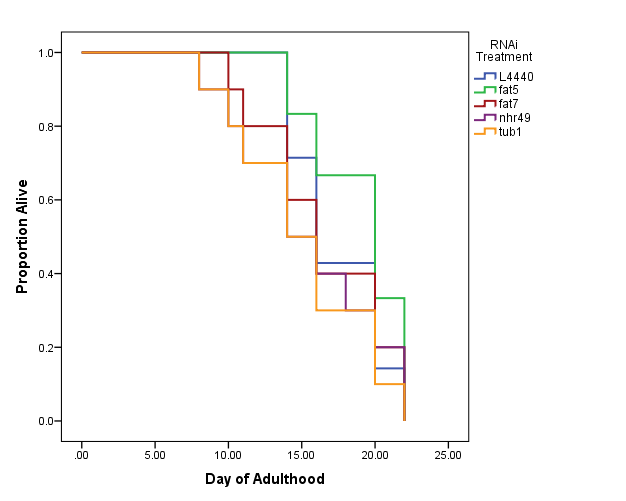


Supplemental Figure 3. Representative Kaplan-Meir survival curve for wild-type alpha-synuclein overexpression strain (JVR208) treated with RNAi treatments : *L4440, fat-5, fat-7, nhr-49 and tub-1* . There was no significant difference in lifespan extension (p > 0.05; Kaplan–Meier log-rank test) between the groups . Each experiment was repeated at least thrice (n=44 animals per treatment group).


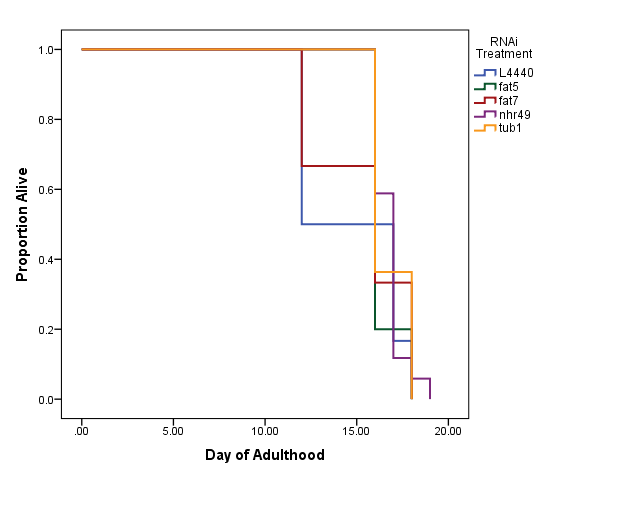


Supplemental Figure 4 Representative Kaplan-Meir survival curve for mutant A53T alpha-synuclein overexpression strain (JVR203) treated with RNAi treatments : *L4440, fat-5, fat-7, nhr-49 and tub-1* . There was no significant difference in lifespan extension (p > 0.05; Kaplan–Meier log-rank test) between the groups . Each experiment was repeated at least thrice (n=44 animals per treatment group).


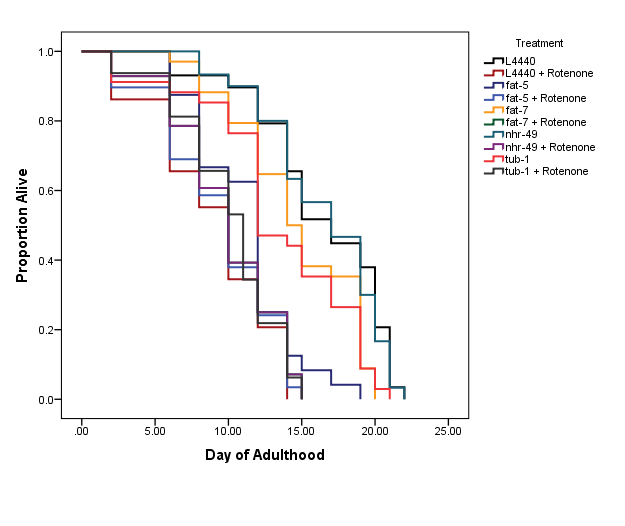


Supplemental Figure 5. Representative Kaplan-Meir survival curve for wild-type strain (TG2435) treated with RNAi treatments : *L4440, fat-5, fat-7, nhr-49* and *tub-1* and with rotenone (0 and 4 μM) . There was significant difference in lifespan (p< 0.05; Kaplan–Meier log-rank test) between the rotenone treated groups . Each experiment was repeated at least thrice (n=44 animals per treatment group).
